# Combined influence of epoch length, cut-point and bout duration on accelerometry-derived physical activity

**DOI:** 10.1186/1479-5868-11-34

**Published:** 2014-03-10

**Authors:** Mark Orme, Katrien Wijndaele, Stephen J Sharp, Kate Westgate, Ulf Ekelund, Soren Brage

**Affiliations:** 1 Medical Research Council Epidemiology Unit, Institute of Metabolic Science, University of Cambridge, Addenbrookes Hospital, Box 285, Cambridge, CB2 0QQ, UK; 2 School of Sport, Exercise and Health Sciences, Loughborough University, Leicestershire, UK; 3 Department of Sports Medicine, Norwegian School of Sport Sciences, Oslo, Norway

**Keywords:** Moderate-to-vigorous, Adults, Measurement, Wear-time, Actigraph, Objective

## Abstract

**Background:**

It is difficult to compare accelerometer-derived estimates of moderate-to-vigorous physical activity (MVPA) between studies due to differences in data processing procedures. We aimed to evaluate the effects of accelerometer processing options on total and bout-accumulated time spent in MVPA in adults.

**Methods:**

267 participants from the ProActive Trial provided 1236 days of valid physical activity (PA) data, collected using a 5-s epoch with ActiGraph GT1M accelerometers. We integrated data over 5-s to 60-s epoch lengths (EL) and applied two-level mixed effects regression models to MVPA time, defined using 1500 to 2500 counts/minute (cpm) cut-points (CP) and bout durations (BD) from 1 to 15 min.

**Results:**

Total MVPA time was lower on longer EL and higher CP (47 vs 26 min/day and 26 vs 5 min/day on 1500 vs 2500 cpm on 5-s and 60-s epoch, respectively); this could be approximated as MVPA = exp[2.197 + 0.279*log(CP) + 6.120*log(EL) - 0.869*log(CP)*log(EL)] with an 800 min/day wear-time. In contrast, EL was positively associated with time spent in bout-accumulated MVPA; the approximating equation being MVPA = exp[54.679 - 6.268*log(CP) + 6.387*log(EL) - 10.000*log(BD) - 0.162*log(EL)*log(BD) - 0.626*log(CP)*log(EL) + 1.033*log(CP)*log(BD)]. BD and CP were inversely associated with MVPA, with higher values attenuating the influence of EL.

**Conclusions:**

EL, CP and BD interact to influence estimates of accelerometer-determined MVPA. In general, higher CP and longer BD result in lower MVPA but the direction of association for EL depends on BD. Reporting scaling coefficients for these key parameters across their frequently used ranges would facilitate comparisons of population-level accelerometry estimates of MVPA.

## Background

Regular moderate-to-vigorous physical activity (MVPA) is beneficial for preventing non-communicable diseases [1]. Although the health benefits of participation in MVPA have been well documented, population levels are believed to be relatively low [2,3]. Despite extensive research interest in physical activity (PA), there is limited data at the population level, most of which is obtained in high-income countries using self-report measures [2] which are susceptible to several forms of bias [4].

The use of wearable monitors such as accelerometers for the measurement of PA levels across different intensities in observational and intervention studies has increased over the last decade [5]. However, even with the use of accelerometers, uncertainties remain with regards to interpretation of the data generated. With advances in technology, variability in data collection settings has increased, presenting some challenges for comparisons between studies and with historical data.

Accelerometers were historically most often set-up to record body movement at 60-s epochs [6], as this sampling frequency permitted data collection for a longer duration compared with shorter epoch lengths with available instruments [7]. Some studies have investigated the effect of epoch duration on measured time spent in different PA intensities in children [8] and the consensus is that shorter epochs should be used to obtain a more accurate representation of young people’s PA levels [9]. This may well hold true for adults as well, but to the best of our knowledge, there is only limited evidence on the effects of epoch length in adults. A study by Gabriel et al. [10] compared 10-s and 60-s epochs in overweight, postmenopausal women and found higher total MVPA time when data were presented in 10-s epochs. However, given the homogenous sample used in the study, further research is needed. So far, the majority of studies have examined habitual PA using 60-s epochs in healthy adults, and it is therefore unclear how shorter epoch length settings might influence estimates of MVPA in adults.

Another issue is the selection of cut-points for classification of intensity levels, which has obvious implications for estimating the proportion of time spent at various intensities. Matthew [11] demonstrated the considerable variety of cut-points that have been used, especially for MVPA. The two cut-points predominantly used in adults to determine MVPA are 1952 counts-per-minute (cpm) for the Actigraph accelerometer derived from the relationship between counts and energy expenditure during treadmill walking and jogging [12], and 2020 cpm first used in NHANES derived from a weighted average of all published data available at the time of analysis [13]. Researchers may wish to compare findings from studies using these cut-points but with limited evidence to support their comparability, illustrating the need to investigate the impact of applying different cut-points, preferably in conjunction with a range of epoch lengths, on time spent in MVPA.

Variability in the assessment of MVPA is not only restricted to epoch and cut-point settings. Interpretation of how MVPA can be accumulated in bouts of varying duration can also be brought into question. Current MVPA guidelines [14] suggest that bouts of ≥10 mins are required for health benefits. Most studies investigating bout-accumulated MVPA defined a bout as ≥10 mins of continuous activity above the cut-point threshold [15-17]. Ayabe et al. [18] investigated bout durations of <32-s, >1 min, >3 mins and >5 mins in relation with health outcomes. However, there is limited evidence on the influence of allowing shorter or longer bout lengths, and the interaction with cut-point and epoch lengths, on time spent in MVPA in adults.

We aimed to describe the scaling effects of epoch length and cut-point selection on time spent in total MVPA, and also on bout-accumulated MVPA including the effect of bout duration.

## Methods

### Data

Data from the third wave of measurement of the ProActive UK Trial were used for these analyses (n = 270). In brief, the aim of ProActive was to assess the efficacy of a behavioural intervention designed to increase PA levels among adults at high risk of type 2 diabetes. Full details of the study can be found elsewhere [19]. Body mass index was derived from objective measures of height and weight. Bio-electrical impedance (Bodystat, Isle of Man, UK) was used to estimate fat mass and fat-free mass. Ethical approval was obtained from the Eastern Multi-Centre Research Ethics Committee, and West Suffolk, Cambridge, Huntingdon and West Essex Local Research Ethics Committees and participants provided written informed consent.

### Assessment of free-living PA

The GT1M Actigraph (ActiGraph, LLC, Pensacola, FL) uniaxial accelerometer was used to measure participants’ physical activity. The accelerometers were initialised to collect data in 5-s epochs, worn on an elastic belt around the waist for 5 days, and downloaded using ActiLife version 4.3.0 (ActiGraph, LLC, Pensacola, FL).

### Data processing

The accelerometer files were processed using KineSoft version 3.3.63 (KineSoft, Saskatchewan, SK, Canada). For the purpose of this investigation, copies of the original files were made, and re-integrated into 10-s, 15-s, 20-s, 30-s and 60-s epochs resulting in six copies of each record differentiated only by epoch length. The range and number of epoch lengths investigated increase the power of the regression models to establish scaling coefficients and cover frequently used epoch lengths.

Non-wear time was defined as continuous runs of zeros lasting ≥60 consecutive mins, with no allowance for counts greater than zero. Participants removed the monitor overnight and wear time was defined by subtracting non-wear time (including sleep) from 24 h. Data were included if participants accumulated a minimum of 500 mins (8 h 20 mins) of valid activity recordings per day for at least 1 day.

Of the original sample of 270 participants with accelerometry data, two accelerometers were set up in 60-s epochs, thus preventing the investigation of shorter epoch lengths. One other accelerometer file was also excluded from further analyses due to failure of meeting the valid day criterion. The analysed sample therefore consisted of 267 individuals. To maximise estimation accuracy, daily data was utilised resulting in 1236 valid days of accelerometer output.

### Statistical analyses

All statistical analyses were performed using STATA 12.0 (StataCorp, College Station, TX). Characteristics of the study sample were described using means and standard deviations. Mean time spent in both total and bout-accumulated MVPA were presented by epoch length and cut-point. To enable examination of curvilinear relationships between accelerometer parameters and MVPA, all variables were first log-transformed (natural). All models were adjusted for wear time (truncated to ≥500 min/day), as individuals wearing an accelerometer for longer each day have a greater opportunity to accumulate MVPA.

The influences of epoch length (5-s, 10-s, 15-s, 20-s, 30-s and 60-s) and MVPA cut-point (1500 cpm to 2500 cpm in intervals of 100 cpm) on mean total time spent in MVPA (natural log-transformed) were estimated using a 2-level mixed effects regression model, with days of measurement at level 1 nested within each participant at level 2. The effect of each covariate was estimated first by including each one in a separate model, and then including all covariates together. Cut-point values were rescaled proportionally for data reintegration in 5-s, 10-s, 15-s, 20-s, and 30-s epochs to calculate time spent engaging in MVPA (e.g. a 2000 cpm cut-point would be rescaled to a cut-point of 1000 counts per 30-s epoch). The interaction term between cut-point and epoch length (both log-transformed) was also included.

Bout-accumulated time spent in MVPA was defined as continuous activity lasting more than a certain duration above the cut-point threshold. The influences of bout duration (60-s, 120-s, 180-s, 300-s, 600-s and 900-s) on total bout-accumulated time spent in MVPA was then investigated, in conjunction with the effects of epoch length (5-s, 15-s, 30-s and 60-s) and cut-point (1500 cpm, 2000 cpm and 2500 cpm), using a similar method to that described above, and including all 2-way interactions.

## Results

The mean wear time for this population was 842 mins per day with 4.6 valid days per participant, on average. Characteristics of the sample are shown in Table 1. The final sample comprised of 153 women and 114 men with a mean (SD) age of 48.2 (6.2) and 47.4 (5.6) years, respectively. Overall, 41% and 35% of the participants were classified as overweight and obese, respectively.

**Table 1:** Descriptive characteristics of the study sample (n = 267)

	**Women**		**Men**		**All**	
	**n**	**Mean ± SD**	**n**	**Mean ± SD**	**n**	**Mean ± SD**
Age (yr)	153	48.2 ± 6.2	114	47.4 ± 5.6	267	47.9 ± 6.0
BMI (kg · m^2^)	153	29.0 ± 5.6	114	28.7 ± 4.5	267	28.9 ± 5.2
Fat free mass (kg)	142	48.2 ± 6.0	103	67.9 ± 9.5	245	56.5 ± 12.4
Fat mass (kg)	142	28.3 ± 10.9	103	24.3 ± 8.8	245	26.6 ± 10.3

### Total time spent in MVPA

Wear time was strongly associated with total time spent in MVPA, for example person-days with a wear time of 600 mins contained 40.1% (13mins 27 s) less MVPA than those with 800 mins for an epoch length of 5-s and a cut-point of 2020 cpm. All models therefore included adjustment for wear time. The influence of epoch length (5-s to 60-s), cut-point (1500 cpm to 2500 cpm) and wear time (≥500 mins) on mean total time spent in MVPA could be estimated using the following equation:

$$\begin{array}{ll} \mathrm{TotalMVPA}\;\left(\mathrm{mins}\right)= & \exp[0.279*\log\left(\mathrm{CutPoint}\right)+6.120\\ & *\log\left(\mathrm{EpochLength}\right)-0.869\\ & *\log\left(\mathrm{CutPoint}\right)*\log\left(\mathrm{EpochLength}\right)\\ & +\;1.782*\log\left(\mathrm{WearTime}\right)-9.715] \end{array}$$

All coefficients in this model are significant at p ≤ 0.004 (Additional file 1: Table S1). Over and above the variance in total MVPA accounted for by wear time (4%), cut-point and epoch length each explained about 8% of variance, with their interaction adding another 0.9%.

**Figure 1:**
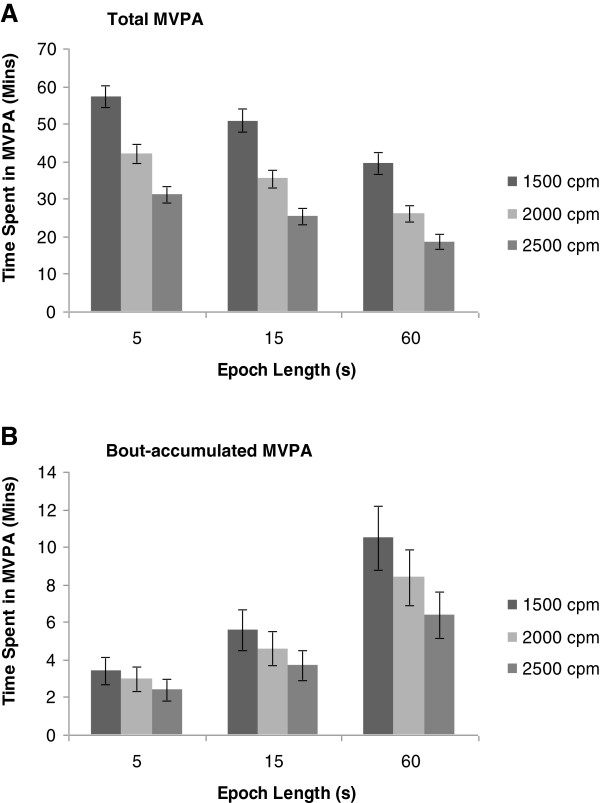
**Mean time spent in total and bout-accumulated moderate-to-vigorous physical activity, by epoch length.** Legend; Total time spent in moderate-to-vigorous physical activity (MVPA) is represented in panel **A** and bout-accumulated (>10 min) time spent in MVPA in panel **B**. For illustrative purposes, three epoch lengths (5-s, 15-s and 60-s) are chosen to represent the range of epoch lengths analysed, with three cut-points (1500 cpm, 2000 cpm and 2500 cpm) displayed for each epoch. Values are means with 95% confidence intervals (error bars) of empirical data, estimated from ANOVA repeated measures.

Total MVPA time was inversely associated with both epoch length and cut-point but the effects are somewhat stronger at lower cut-points and shorter epochs, as indicated by the opposite sign interaction term and shown in Figure 1 panel A. Comparing the commonly used 1952 and 2020 cpm cut-points, there was a significant difference in total time spent in MVPA of 4.1% (1 min 47 s; 95% CI = 6 s to 3mins 30s) for the 5-s epoch length. This difference was similar but not statistically significant for 15-s epochs (4.5%, 1 min 39 s; 95% CI = 0 s to 3mins 20s) nor for 60-s epochs (4.8%, 1 min 17 s; 95% CI = 20s to 2mins 54 s). The effect of re-integrating 5-s to 60-s epochs was a decrease in MVPA of 38.3% (15mins 52 s; 95% CI = 14mins 6 s to 17mins 24 s) compared with 15.8% (6mins 33 s; 95% CI = 4mins 54 s to 8mins 12 s) and 26.7% (9mins 19 s; 95% CI = 7mins 36 s to 10mins 54 s) for 5-s to 15-s and 15-s to 60-s reintegration, respectively. These differences by epoch length using the 2020 cpm cut-point were similar when using the 1952 cpm MVPA threshold.

### Bout-accumulated time spent in MVPA

The influence of bout duration (60-s to 900-s), epoch length (5-s to 60-s), cut-point (1500 cpm to 2500 cpm) and wear time (≥500 mins) on mean bout-accumulated time spent in MVPA can be described using the following equation:

$$\begin{array}{ll} \mathrm{Bout}-\mathrm{accumulated}\;\mathrm{MVPA}\;\left(\mathrm{mins}\right)= & \exp[-\;6.268*\log\left(\mathrm{CutPoint}\right)\\ & +\;6.387*\log\left(\mathrm{EpochLength}\right)\\ & -\;10.000*\log\left(\mathrm{BoutDuration}\right)\\ & -\;0.162*\log\left(\mathrm{EpochLength}\right)\\ & *\log\left(\mathrm{BoutDuration}\right)-\;0.626\\ & *\log\left(\mathrm{CutPoint}\right)*\log\left(\mathrm{EpochLength}\right)\\ & +\;1.033*\log\left(\mathrm{CutPoint}\right)\\ & *\log\left(\mathrm{BoutDuration}\right)+2.147\\ & *\log\left(\mathrm{WearTime}\right)+40.327] \end{array}$$

All coefficients in this model are significant at p < 0.001 (Additional file 1: Table S2). Bout duration accounted for 38% of the variance in bout-accumulated MVPA over and above that explained by wear time (0.5%), with epoch length and cut-point jointly adding 4% and the three 2-way interaction terms adding another 0.7%.

There was no significant difference (~3%) in time spent in bout-accumulated MVPA between 1952 cpm and 2020 cpm for all epochs (5-s: 7 s, 95% CI = -42 s to 30s; 15-s: 8 s, 95% CI = -54 s to 36 s; 60-s: 16 s, 95% CI = -1 min 24 s to 48 s). Figure 1 panel B demonstrates the reverse epoch length effect on the time spent in bout-accumulated MVPA (≥2000 cpm; ≥10 mins) compared with total time in this intensity. The effect of converting 5-s to 60-s epochs was a gain in bout-accumulated MVPA of 64.4% (5mins 19 s; 95% CI = 4mins 24 s – 6mins 12 s) compared with 35.1% (1mins 35 s; 95% CI = 42 s – 2mins 30s) and 45.2% (3mins 44 s; 95% CI = 2mins 54 s – 4mins 36 s) for 5-s to 15-s and 15-s to 60-s reintegration, respectively. Bout duration significantly impacts upon time spent in MVPA (≥2000 cpm; 60-s). Analysing bout-accumulated time spent in MVPA using 10 min bouts resulted in 68.1% less time in MVPA, compared to a 1 min bout length (17mins 42 s; 95% CI = -16mins 18 s – 19mins), and a 33.6% reduction versus 5 min bouts (4mins 12 s; 95% CI = -2mins 54 s – 5mins 36 s). Using 1 min bouts resulted in a positive difference in MVPA of 51.9% (13mins 30s; 95% CI = 12mins 6 s – 14mins 48 s), compared with a 5 min bout length.

**Figure 2:**
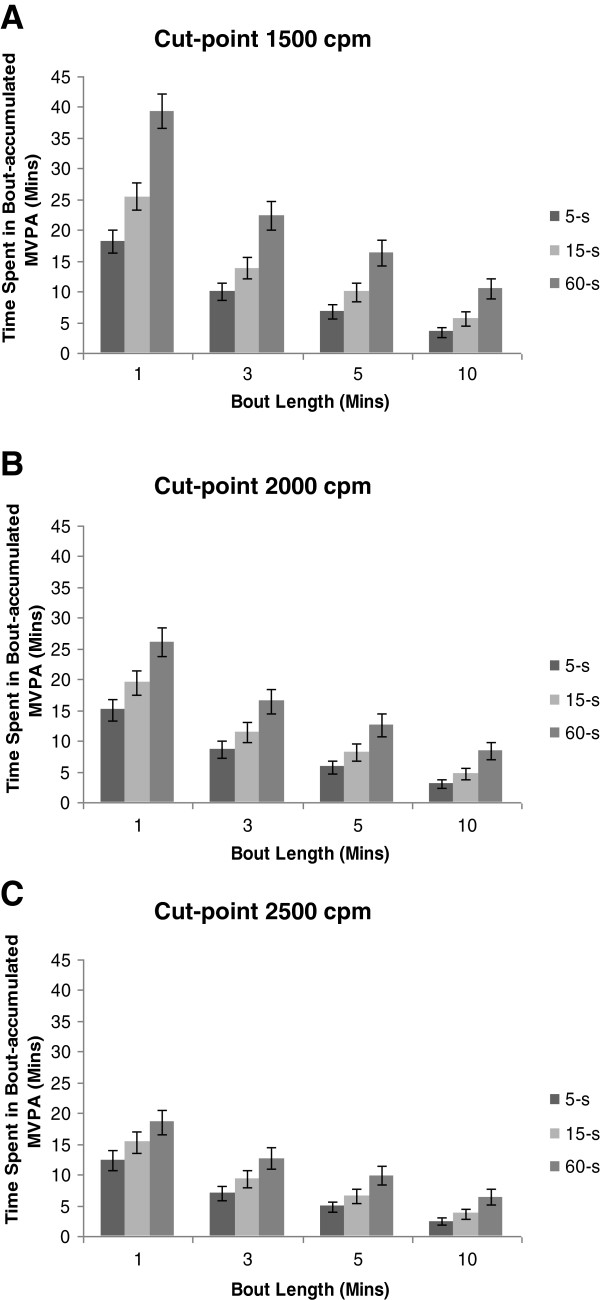
**Mean time spent in bout-accumulated moderate-to-vigorous physical activity, by bout duration.** Legend; Moderate-to-vigorous physical activity (MVPA) cut-point threshold of 1500 cpm is shown in panel **A**, 2000 cpm in panel **B**, and 2500 cpm in panel **C**. For each panel, bout durations of ≥1 min, ≥3 mins, ≥5 mins and ≥10 mins are given, with three epochs (5-s, 15-s and 60-s) for each bout criterion. Values are means with 95% confidence intervals (error bars) of empirical data, estimated from ANOVA repeated measures.

Figure 2 highlights the influence of bout duration on time spent in MVPA, and the interactions associated with cut-point and epoch length. To summarise, time spent in bout-accumulated MVPA is inversely associated with bout duration and cut-point. There is a positive relationship with epoch length for time spent in bout-accumulated MVPA with significant interaction, so that epoch length has a larger influence on time spent in MVPA when shorter bout durations are examined. Increasing bout duration reduces time spent in MVPA, with the effect attenuated by higher cut-point thresholds and epoch lengths. The mean effect of selecting a 10 min bout duration (2000 cpm; 60-s) is a decrease in time spent in MVPA of 49.4% (8mins 6 s; 95% CI = 6mins 42 s – 9mins 24 s) compared with 3 min bouts. For bouts of at least 10 mins (2000 cpm), integrating epoch length from 5-s to 60-s increased time spent in MVPA by 276% (5mins 18 s; 95% CI = 4mins 30s – 6mins 18 s). Increasing the cut-point from 1500 cpm to 2500 cpm (10 min bouts; 60-s) decreases time spent in bout-accumulated MVPA by 48.5% (4mins 0 s; 95% CI = 2mins 54 s – 5mins 6 s).

## Discussion

We investigated the combined influences of accelerometer processing parameters across a range of epoch lengths, cut-points and bout durations on time spent in total and bout-accumulated MVPA in adults. Our findings highlight the need to make informed decisions concerning the processing and interpretation of accelerometer data. The regression models reveal how time spent in total and bout-accumulated MVPA are affected by epoch length, cut-point and bout duration.

The standardisation of accelerometer-derived PA measurement is a challenge within the field of objective monitoring. We have demonstrated that the data collection and processing decisions contribute, both as independent and interacting factors, to the estimated amount of time spent in MVPA within a given population. Our analyses provide insight into how participants tend to accumulate their activity. For example, if total and bout-accumulated time spent in MVPA is very different using shorter epochs, as is the case in this current investigation, it can be concluded that participants accumulate their MVPA in a relatively sporadic nature. We therefore recommend that these analyses are conducted for other populations, providing information on the effects of these parameters.

Epoch length and bout duration were found to interact; there is a reverse association for epoch length between total and bout-accumulated MVPA. Longer epochs result in greater accumulation of MVPA in bouts due to a buffering effect, which results in a reduced likelihood of bout termination by short interruptions. Conversely, shorter epochs are more likely to end bouts of MVPA. The effect of longer epochs on bout-accumulated time spent in MVPA is attenuated by the use of higher cut-point thresholds or longer bout durations. We used a continuous bout definition in the present investigation and did not include the concept of bout interruption allowances [15,20]. It is a matter of definition if for example a 21-min segment of MVPA with an interruption of 1 min in the middle is considered a single bout of 20 mins or two bouts of 10 mins.

Gabriel et al. [10] found more than 15 mins difference in total time spent in MVPA between 60-s and 10-s epochs in female adults which is comparable with the results of this study in men and women. Caution must therefore be given to comparisons between adult PA investigations using accelerometry without adjustments for epoch length, as these differences are likely to impact on adherence to PA guidelines and associations with clinical outcomes. As suggested by Gabriel et al. [10], intermittent MVPA is likely to be misclassified as light intensity when summed over a full minute. It is therefore not only the sporadic nature of children’s activity [9] but also the intermittent activity of adults which makes epoch length a significant factor on the interpretation of accelerometer data.

Nilsson et al. [8] investigated the role of epoch length in children, demonstrating that the magnitude of the epoch effect is amplified among individuals with higher activity levels. In children, this was predominantly seen in the vigorous and very vigorous intensity categories and only to a lesser degree in moderate intensity. It seems likely, based on both total and bout-accumulated time spent in MVPA and previous findings [10], that this amplification is also present in adults but the effect is shifted towards less vigorous intensities.

The finding that a longer epoch length results in increased bout-accumulated time spent in MVPA is supported by Nilsson et al. [8]. For continuous 10 min bouts of MVPA, large differences were found between 5-s, 15-s and 60-s epochs, as few daily activities require moderate intensity sustained for at least 10 consecutive mins. Using a 60-s epoch allows activity to drop below the threshold of MVPA temporarily, as counts are summed over the minute. Shorter epochs will capture this instance of lower intensity, thus the observed difference between total and bout-accumulated MVPA will depend on the relative proportion of MVPA undertaken in steady-state for any given population. As our results show, this epoch influence becomes important relative to the selection of shorter or longer bout durations. This interaction between epoch length and bout duration on MVPA should be considered when comparing bout and non-bout MVPA estimates.

The extent to which epoch length impacts on bout-accumulated MVPA serves to highlight an important issue arising in the field of accelerometry. There is a growing interest in the field with regard to the accumulation of MVPA in bouts. However, it is governed more severely by epoch length compared to total time spent in MVPA. Consequently the processing of bouts using shorter epochs, although more accurately detecting lapses in activity, may not reflect the underpinning physiology. In parallel to developing appropriate definitions and methods for determining bout-accumulated MVPA using shorter epochs, further research into the health effects of varying bout durations, and the allowance of interruptions within them, is required.

The comparison between 1952 cpm [12] and 2020 cpm [13] cut-points on total and bout-accumulated time spent in MVPA found the two sets of cut-points to be reasonably comparable across epochs. Fewer than two minutes difference (~5%) between the cut-points was observed in all epochs for both total and bout-accumulated MVPA, thus this difference is unlikely to greatly influence the conclusions of studies assuming their comparability. Cut-point values which are more distant on the movement intensity continuum, however, result in significant differences in time spent in MVPA as also observed in other populations; for example every 500 cpm increase in cut-point between 1000 cpm to 2000 cpm resulted in a 50% reduction of MVPA in older American adults taking part in NHANES 2003–6 (60-sec epochs), a pattern which was consistent across age decades despite differences in absolute levels [21]. The cut-point also interacts with epoch length in determining the likelihood of counts averaging above the chosen MVPA threshold. The effect of selecting a higher cut-point diminishes bout-accumulated MVPA to a lesser degree with longer bout durations.

Wear time was found to be associated with time spent in MVPA. It appears that wear time bias or selection effects may exist in relation to time spent in MVPA. However, the extent to which wear time contributes to the overall effects of accelerometer processing criteria may depend on multiple factors, including the activity level of the population. It may be that those who do more MVPA are more likely to wear the monitor for longer on average each day. It could also be that wearing the accelerometer for longer simply provides greater opportunity to accumulate MVPA. This finding adds to that of Pettee Gabriel et al. [22] and Schmidt et al. [23] linking wear time with sedentary behaviours. It is therefore recommended that researchers not only specify non-wear detection criteria for the time-series processing (e.g. zero-string length) and minimum wear time criteria, including monitored days, but also report the (truncated) mean wear time of the sample and how it influences the activity outcome in question.

This study highlights the need for researchers to consider sample characteristics when investigating bout accumulated MVPA. In populations such as ProActive, time spent in MVPA differs substantially when bouts are introduced. The ProActive cohort is relatively inactive; of the 98,556 days of data analysed (differentiated by different settings of epoch length, cut-point or bout length for each participant), 48,581 days (49.3%) contained no MVPA whatsoever and 64,636 days (65.6%) had fewer than 10 mins of bout-accumulated MVPA.

There are important limitations to consider when interpreting the results of the current investigation. We only examined the influence of epoch in the 5-s to 60-s range, the effect of MVPA cut-point in the 1500 cpm to 2500 cpm range, and the effect of bout duration in the 1 min to 15 min range; it is unlikely that the established relationships hold true outside those ranges. In addition, the relatively small sample size means analyses using larger, more heterogeneous populations are needed to establish the generalizability of these findings. However, the relative inactivity of this population means the estimated scaling effects of these processing criteria are likely to be conservative.

Technological advancements in accelerometry have increased the capabilities of available data collection, processing methods and interpretation of activity estimates. Accelerometers are now capable of collecting data using 1-s epochs or even raw acceleration. This can then be used in conjunction with emerging signal processing and pattern recognition models such as artificial neural networks [7,24] to infer activity type and potentially also enhance the assessment of MVPA. Despite these advances, the issues raised in the current study will remain applicable as the challenge of developing standardised methods of categorising population activity levels using accelerometry will still exist. The advancements in accelerometer technology means researchers are faced with a large number of decisions with regards to the processing and interpretation of PA data. Cut-point selection, epoch length and bout duration have all been identified as influencing MVPA outcomes. We would encourage researchers to collect PA data in the highest available resolution and replicate the analyses of the current investigation to establish regression coefficients for these three parameters in other populations. This enables greater understanding of how populations accumulate activity of a particular intensity, duration and frequency. The present analyses also highlight the need for investigations into the physiological impact of bout-accumulated MVPA in parallel to advances in accelerometer processing.

## Conclusions

In conclusion, methodological issues with regards to the processing and interpretation of accelerometer-derived time spent in MVPA in adults have been identified. The selection of epoch length, cut-point threshold and bout duration influences MVPA outcomes as independent and interacting parameters. It is recommended that researchers use the highest accelerometer resolution available in order to maximise the opportunities for summarising the data appropriately with respects to epoch length, cut-point and bout duration in comparisons with existing population data. When this is not directly possible, the description of population levels of MVPA across a range of these settings allows for a more standardised methodology for accelerometer-based PA.

## Abbreviations

BD: Bout duration; cpm: Counts/minute; CP: Cut-point; EL: Epoch length; MVPA: Moderate-to-vigorous physical activity; PA: Physical activity.

## Competing interests

None of the authors have any financial or non-financial conflicts of interest to declare.

## Authors’ contributions

MO drafted the manuscript and is guarantor. SB conceived the study question. MO, KWe, and UE acquired and processed the raw accelerometer data. MO and SB analysed the data. MO, KWi, SJS and SB interpreted data. All authors revised the article for important intellectual content and approved the final version of the manuscript.

## Supplementary Material

Additional file 1: Table S1 Beta coefficients with 95% confidence intervals for total (non-bout) accumulated MVPA. **Table S2**: Beta coefficients with 95% confidence intervals for bout-accumulated MVPA. Click here for file
