# Supplementary material for: Combined influence of epoch length, cut-point and bout duration on accelerometry-derived physical activity
Source: Int J Behav Nutr Phys Act. 2014 Mar 10;11:34. doi: 10.1186/1479-5868-11-34 (PMC4008000; doi:10.1186/1479-5868-11-34)
Supplement: Additional file 1: Table S1 — Beta coefficients with 95% confidence intervals for total (non-bout) accumulated MVPA. Table S2: Beta coefficients with 95% confidence intervals for bout-accumulated MVPA. [file 1479-5868-11-34-S1.docx]

|  | *Βeta coefficient* | *95% CI* | |
| --- | --- | --- | --- |
| ln Wear time | 1.782 | -0.932 | -0.805 |
| ln Cut-point | 0.279 | 0.090 | 0.467 |
| ln Epoch Length | 6.120 | 5.637 | 6.602 |
| INTERACTION, ln Cut-point * ln Epoch Length | -0.869 | -0.933 | -0.805 |
| Intercept | -9.715 | -11.191 | -8.240 |

**Supplementary table 1:** Beta coefficients with 95% confidence intervals for total (non-bout) accumulated MVPA

Data are beta coefficients with 95% confidence intervals for log-transformed MVPA time.

**Supplementary table 2:** Beta coefficients with 95% confidence intervals for bout-accumulated MVPA

|  | *Βeta coefficient* | *95% CI* | |
| --- | --- | --- | --- |
| ln Wear time | 2.147 | 2.007 | 2.287 |
| ln Cut-point | -6.268 | -6.984 | -5.552 |
| ln Epoch Length | 6.387 | 5.525 | 7.248 |
| ln Bout Duration | -10.000 | -10.835 | -9.165 |
| INTERACTION, ln Cut-point * ln Epoch Length | -0.626 | -0.738 | -0.513 |
| INTERACTION, ln Cut-point * ln Bout Duration | 1.033 | 0.924 | 1.143 |
| INTERACTION, ln Epoch Length * ln Bout Duration | -0.162 | -0.185 | -0.138 |
| Intercept | 40.327 | 34.798 | 45.856 |

Data are beta coefficients with 95% confidence intervals for log-transformed MVPA time in bouts.
